# Reconstructing spectral cues for sound localization from responses to rippled noise stimuli

**DOI:** 10.1371/journal.pone.0174185

**Published:** 2017-03-23

**Authors:** A. John Van Opstal, Joyce Vliegen, Thamar Van Esch

**Affiliations:** Radboud University/Department of Biophysics, HG00.831//Donders Center for Neuroscience/Heyendaalseweg 135, 6525 AJ Nijmegen, the Netherlands; Tokai University, JAPAN

## Abstract

Human sound localization in the mid-saggital plane (elevation) relies on an analysis of the idiosyncratic spectral shape cues provided by the head and pinnae. However, because the actual free-field stimulus spectrum is a-priori unknown to the auditory system, the problem of extracting the elevation angle from the sensory spectrum is ill-posed. Here we test different spectral localization models by eliciting head movements toward broad-band noise stimuli with randomly shaped, rippled amplitude spectra emanating from a speaker at a fixed location, while varying the ripple bandwidth between 1.5 and 5.0 cycles/octave. Six listeners participated in the experiments. From the distributions of localization responses toward the individual stimuli, we estimated the listeners’ spectral-shape cues underlying their elevation percepts, by applying maximum-likelihood estimation. The reconstructed spectral cues resulted to be invariant to the considerable variation in ripple bandwidth, and for each listener they had a remarkable resemblance to the idiosyncratic head-related transfer functions (HRTFs). These results are not in line with models that rely on the detection of a single peak or notch in the amplitude spectrum, nor with a local analysis of first- and second-order spectral derivatives. Instead, our data support a model in which the auditory system performs a cross-correlation between the sensory input at the eardrum-auditory nerve, and stored representations of HRTF spectral shapes, to extract the perceived elevation angle.

## Introduction

Human directional hearing relies on the processing of acoustic cues that originate from the interaction of sound waves with the head and pinnae. Sound localization in the horizontal plane (*azimuth*) utilizes binaural differences in sound arrival time and phase for frequencies up to about 1.5 kHz, and in sound level for the higher frequencies [1].

For localization in the vertical plane (*elevation*), the auditory system employs the fact that sound waves (above 3–4 kHz) arriving at the ears are reflected and diffracted within the asymmetrical pinna aperture before reaching the eardrum, which results in an elevation-dependent pattern of amplifications and attenuations of the amplitude spectrum in the ear canal [2], [3]. These patterns are known as Head Related Transfer Functions, or HRTFs (e.g. [4]), and the auditory system has to extract the sound-source elevation angle from these spectral shape cues (see [1], and [5], for reviews). It is generally assumed that the system has acquired, and stored, knowledge about the HRTFs through learning and interacting with the acoustic environment. Indeed, studies by Hofman et al. [6] and by Van Wanrooij and Van Opstal [7], in which the pinna geometry was altered by inserting a small mold in the pinna concha, showed that the human auditory system can learn new sets of HRTFs within one to a few weeks. The auditory system also adapts to the slow pinna growth throughout our life span [8]. Although the driving force for this learning is yet to be established, it is likely to be guided by feedback from the environment, e.g. by combining information about self motion (head and body movements), and from the visual system, with the acoustic input and the associated sound-localization errors (e.g. [9]).

In this paper, we study the mechanisms that may underly the neural mapping from the spectral shape cues into an estimate of a sound’s elevation.

Note, that the auditory system faces a fundamental problem in determining the elevation angle of the sound source, *ε*_*s*_, as the acoustic pressure at the eardrum, *s*(*t*; *ε*_*s*_) (here denoted as the *sensory signal*), results from a convolution of the sound-source pressure in the free field, *x*(*t*), the direction-dependent acoustic filter of the head and pinna, *h*_pinna_(*t*; *ε_s_*), and the (direction-independent) filtering provided by the ear canal, *h*_canal_(*t*):

$$s\left(t;\varepsilon_{s}\right)=h_{\text{canal}}\left(t\right)⋆h_{\text{pinna}}\left(t;\varepsilon_{s}\right)⋆x\left(t\right)$$ (1)

where ⋆ indicates convolution. Fourier transformation of Eq 1, followed by taking the logarithm of the amplitude spectrum and frequency results in a spectral representation of the sensory signal, as it is thought to be represented in the auditory system:

$$\log S\left(\omega ;\varepsilon_{s}\right)=\log X\left(\omega \right)+\log H\left(\omega ;\varepsilon_{s}\right)$$ (2)

with *ω* the frequency in octaves, *S*(*ω*; *ε*_*s*_) the sensory spectrum, *X*(*ω*) the sound-source spectrum, and *H*(*ω*; *ε*_*s*_) the combined transfer characteristic of head, pinna, and ear canal (the HRTF; [10]).

Both the sound-source spectrum and the associated HRTF are a-priori unknown to the auditory system, which renders the estimation of sound-source elevation, *ε*_*s*_, on the basis of spectral filtering an ill-posed problem. This entails that infinitely many combinations of sound-source spectra and HTRFs may satisfy Eq 1, and that, as a consequence, a unique solution does not exist. In order to deal with this fundamental problem, the auditory system is thought to make certain assumptions about the sound-source spectrum. Different mechanisms have been proposed in the literature to explain how the elevation angle may be extracted from the sensory input.

**Models for elevation localization.** Essentially, two types of models have been put forward. In the first, the auditory system searches for a particular feature in the sensory spectrum (e.g. a spectral peak, or a notch), which is compared to stored knowledge about the HRTFs. The localization percept is then determined by the HRTF containing that particular feature in its amplitude spectrum. In the second type of model, the entire spectrum is analyzed and compared to the spectral shapes of stored HRTFs.

**The CPA model.** In their search for the spectral features that underly elevation perception, Musicant and Butler [11] found that narrow-band noises are localized on the basis of their central frequency (CF) rather than by their actual location, and that the CF corresponds to an important region of amplification in the HRTF that is associated with the perceived location. This led Butler and colleagues to propose that the peaks in the sensory spectrum act as a natural cue for sound localization. They introduced the concept of the *covert peak area* (CPA; [11], [12], [13], [14]), which is defined as the region in space from which a narrow band of noise generates a maximum sound pressure level at the ear canal entrance. Rogers and Butler [14] bandpass-filtered noise stimuli to contain only frequencies associated with a particular CPA for “down” or “up” locations in the vertical plane for a specific listener. Monaural elevation judgments of the listener were in general agreement with the CPA theory. Moreover, Butler and Musicant [13] also found that for broadband noise stimuli in which selected frequency segments were attenuated, binaural localization judgments were displaced away from the CPAs associated with the attenuated frequency regions. These findings show that energy peaks in the sound spectrum have a large influence on sound localization.

**Cross-correlation models.** A model of the second type was first formulated by Middlebrooks [15], who proposed that to solve the ill-posed problem, the auditory system assumes that spectra of natural sounds are broadband and flat (*X*(*ω*) = constant). In this case, the spectrum at the eardrum is entirely dominated by the HRTF associated with the sound-source direction. In his model, the auditory system performs a cross-correlation between the sensory spectrum and a library of stored broadband HRTFs.

However, the assumption of a flat source spectrum may be too strict for adequate sound-localization performance. Indeed, sound-localization studies indicate that there is a considerable tolerance as to the shape of the amplitude spectrum. For example, Kulkarni and Colburn [16] found that spectral details may not be very important in sound localization; considerable smoothing of the HRTFs is allowed without affecting the perceived elevation. Thus, in their extended formulation of the cross-correlation model, Hofman and Van Opstal [10] demonstrated mathematially that as long as the HRTFs are unique for every elevation angle, and provided that source spectra do not resemble any of the listener’s HRTFs (i.e. the correlation between the source spectrum and the HRTFs is low), the cross-correlation between the sensory spectrum and the total set of HRTFs will be guaranteed to peak at the HRTF of the actual sound direction. Hence, if localization would be based on determining the HRTF of maximum cross-correlation, it will be accurate for a broad class of spectral shapes. Mislocalizations will occur only if the source spectrum does correlate well with one or more HRTFs. Support for this idea was provided by Hofman et al. [6], and more recently by Van Wanrooij and Van Opstal [7], and Bremen et al. [17].

**Local first and second derivative models.** An alternative model that analyzes the entire spectral shape of the sensory spectrum, and that is not restricted to flat source spectra, was proposed by Zakarauskas and Cynader [18]. They hypothesized that as the amplitude spectra imposed by the HRTF are rather steep, the auditory system should have no problem in localizing sounds for which either the source spectrum is locally flat, or the slope of the spectrum is locally constant. They developed two computational models of spectral cue localization that were based on the first and second derivatives of the sensory spectrum. Their computer simulations indicated that a system assuming a source spectrum with a flat second derivative yielded more accurate and robust localization results than the model based on a flat first derivative.

**Testing the different models.** In a previous study, Hofman and Van Opstal [19] performed an experiment in which they exploited the prediction from the cross-correlation model, that if the sound-source spectrum would resemble any of the stored HRTFs, the perceived elevation would be mislocalized in the direction of that HRTF. They presented listeners with a large set of broadband sounds that had randomly shaped amplitude spectra, emanating from a fixed speaker at the straight ahead location. The elevation distributions of eye-movement localization responses for the entire set of rippled-noise stimuli were used to reconstruct the potential spectral features underlying the localization of sound-source elevation.

The reconstruction (details described below, see Methods) was based on a linear weighting of all employed rippled spectra, in which the maximum likelihood estimate of each stimulus served as its weighting factor. Interestingly, the resulting reconstructed spectral shapes appeared to resemble the listeners’ actual HRTFs, and thus seemed to support the hypothesis that perceived sound-source elevation may be determined by the entire spectral shape of the HRTF, rather than by a single prominent spectral feature. However, the experiment was not specifically designed to dissociate the different models described above.

**This study.** In the current paper, we have extended this paradigm, with the aim to test the predictions of the different models. In particular, we have applied different sets of stimuli, in which the shapes of the amplitude spectra were determined by different ripple bandwidths. If a particular spectral feature (like a CPA, or a notch) would determine the elevation percept, the reconstruction of perceived spectral features should yield this particular feature, irrespective of the ripple bandwidth. On the other hand, as the first- and second-order local spectral derivatives of the rippled stimuli systematically varied with ripple bandwidth, the model of Zakarouskas and Cynader [18] predicts that the spatial range of stimulus mislocalizations and ripple bandwidth will covary. Finally, if the reconstructions for the different stimulus sets would turn out to be similar to the spectral shapes of the listener’s HRTFs, and be invariant to the ripple bandwidth, the results would support the spectral cross-correlation model.

Our data show that the psychophysical reconstructions of spectral features yield similar results for the different stimulus sets, and that the distribution of localization responses does not depend in a systematic way on ripple bandwidth. We therefore propose that the auditory system relies on a cross-correlation between the actual sensory spectrum and the set of stored HRTF representations, rather than on a criterion based on local first or second-order spectral derivatives. Because the spatial range of localization responses was strongly influenced by the speaker’s location, our results also indicate that the perceived elevation is not simply determined by the site of maximum cross-correlation.

## Materials and methods

### Generating broad-band rippled noise stimuli

Details on the generation of the random-spectral shape stimuli have been provided in [19]. Briefly, a set of 175 broad-band stimuli was derived from a long array of Gaussian-distributed amplitudes (the ‘root sequence’) that was low-pass filtered at a given bandwidth, here termed the *ripple bandwidth*. A particular stimulus was created from this filtered sequence by selecting a window of 100 samples, representing a random-shaped amplitude spectrum that extended over 3 octaves, from 2.5–20.0 kHz. The windowed stimulus sequence, which served as a filter to create the actual sound, was smoothed by sine-squared on- and offset ramps of 0.5 octave width. The filter was subsequently extended to lower frequencies with a flat 1.0–2.5 kHz band. The resulting function thus represented the rippled stimulus amplitude spectrum, and was subsequently applied to a flat Gaussian white noise sound, cut-off between 1.0 and 20.0 kHz, to generate the actual sound-pressure wave. All stimuli were generated by Matlab software (The Mathworks, Natick, MA, version 12a).

**Fig 1:**
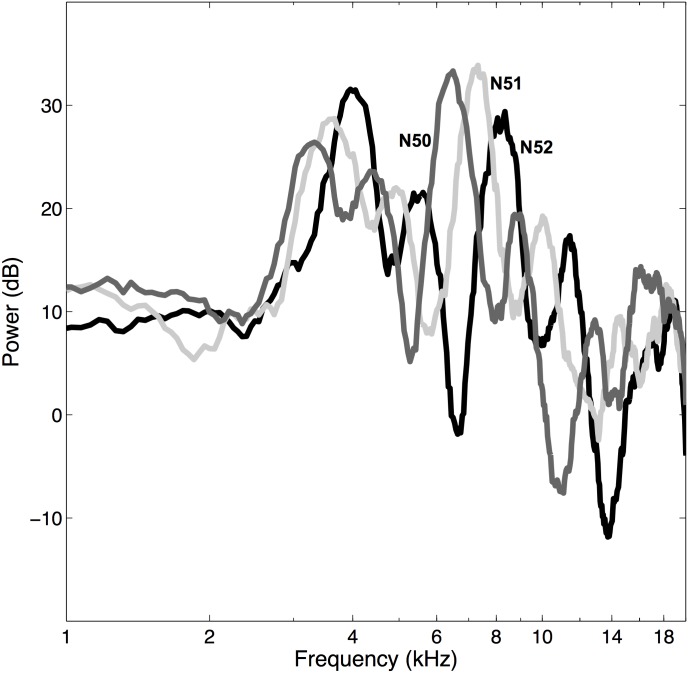
Ripple examples. Three examples of subsequent rippled spectra *X*(*ω*), taken from the 3 cycles/octave (c/o) stimulus set, for stimulus numbers N50, N51, and N52 (in black, light gray, and dark gray respectively). All three spectra are flat from 1–2.5 kHz, and ripples (peak-peak amplitudes up to about 25 dB) run between 3.5–20 kHz. Note the relative shift of the peaks and notches between the subsequent stimulus ranks.

To create the next stimulus in the sequence, the window was shifted across the root sequence by 1/6 octave. In the study of Hofman and Van Opstal [19], the low-pass filter applied to the root sequence had a steep cut-off at 3.0 cycles/octave (c/o). Subsequent stimuli in the set thus had similar shapes, shifted by 1/6 octave. Fig 1 illustrates three typical examples of subsequent random rippled spectra, filtered at 3.0 c/o.

In the present experiments, three different sets of 175 broad-band (1.0–20 kHz) stimuli were generated, with the ripple bandwidths set at 1.5 c/o, 3.0 c/o, and 5.0 c/o, respectively. The stimuli were presented at an intensity of 60 dBA SPL, measured at the site of the listener’s head and had a duration of 250 ms with 5-ms sine-squared on- and offset ramps.

**Fig 2:**
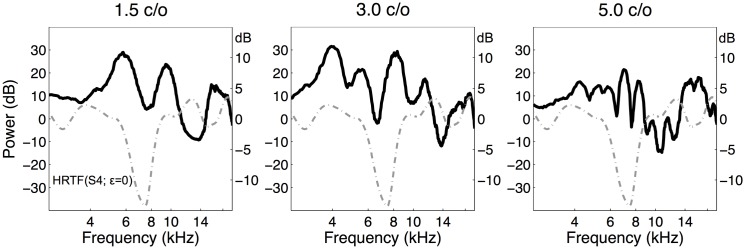
Bandwidth examples. Three representative examples of rippled spectra with a bandwidth of 1.5, 3.0, and 5.0 cycles/octave (c/o; black lines), together with the DTF of 0° elevation of listener S4 (gray dashed line) drawn in the same panels, for comparison. The scale of the DTF is plotted on the right-hand side. The three spectra differ markedly in their amount of spectral variation. Note that the width of the amplitude variations of the DTF appears to fall between those of the 1.5 c/o and the 3.0 c/o stimuli, but that the ripples in the 5 c/o spectrum are much faster.

Examples of representative amplitude spectra from each of the three stimulus sets, are shown in Fig 2, together with a typical HRTF (taken from listener S4, 0° elevation), for comparison. Note that the spectral width of the amplitude variations in the HRTF fall between those of the 1.5 c/o and 3.0 c/o stimuli, but that the variations in the 5 c/o stimulus are clearly much faster.

### Participants

Six listeners (S1–S6) took part in the experiments: four males and two females. Their ages ranged from 22 to 46 years. Three of the listeners were the authors. The other listeners were informed about the actual speaker position, but were kept naive about the purpose of the experiments. All listeners had normal hearing (both audiograms within 20 dBA of audiometric zero, over 0.25–11 kHz), and were experienced in the type of sound-localization experiments carried out in the laboratory.

### Ethics statement

All experimental procedures have been approved by the local ethics committee of the Faculty of Social Sciences of the Radboud University (ECSW, 2016), as they concerned non-invasive observational experiments with healthy adult human subjects. Prior to their participation in the experiments, volunteers gave their written informed consent.

### Experimental setup

Experiments were conducted in a dark and sound-attenuated room with dimensions 3 x 3 x 3 m^3^. The walls, floor, ceiling, and large objects were covered with acoustic foam that effectively absorbed reflections above 500 Hz. The ambient background level in the room was 30 dBA SPL. listeners were seated comfortably on a chair in the center of the room, facing an acoustically transparent thin-wire frontal hemisphere with a radius of 0.85 m, the center of which was approximately aligned with the center of the listener’s head. On this hemisphere 85 red/green light-emitting diodes (LEDs) were mounted at seven eccentricities: *E* = [0, 2, 5, 9, 14, 20, 27, 35]°, relative to straight-ahead ([*E*, Φ] = [0, 0]°) and at twelve directions, given by Φ = [0, 30, …, 330]°, where Φ = 0° is rightward and Φ = 90° is upward. These LEDs were used for calibration of the head-coil measurements and for providing a fixation light at the start of each localization trial.

Sound stimuli were delivered through a broad-range lightweight speaker (Philips AD-44725) mounted on a two-link robot. The robot consisted of a base with two nested L-shaped arms, each driven by a stepping motor (Berger-Lahr VRDM5). To hide the speaker from view, the wire hemisphere was covered with thin black silk.

Two PCs controlled the experiment. One PC-486 was equipped with the hardware for data acquisition (Metrabyte DAS16), stimulus timing (Data Translation DT2817), and digital control of the LEDs (Philips I2C). The other PC-486 generated the acoustic stimuli upon receiving a trigger from the DT2817. The output of this PC was passed through a DA-converter (Data Translation DT2821) at a sampling rate of 50 kHz, fed into a bandpass filter (Krohn-Hite 3343) with a flat passband between 0.2 kHz and 20 kHz, amplified (Luxman A-331), and passed to the speaker. An equalizer (Behringer Ultra-Curve) flattened the speaker transfer characteristic within 5 dB in the passband.

### Measurement of the perceived sound direction

Listeners were told to disregard their knowledge about the actual physical location of the speaker, and were asked to indicate the perceived direction of the sound source by pointing with their head as fast and as accurately as possible. The 2D orientation of the listener’s head was measured with the magnetic search-coil induction technique [20]. Two orthogonal 3x3 m^2^ sets of coils, attached to the walls, floor and ceiling of the room, generated a horizontal (30 kHz) and vertical (40 kHz) oscillating magnetic field. Listeners wore a lightweight helmet, consisting of a narrow strap above the ears, that could be adjusted to fit around the head, and a second strap that ran over the head. A small coil was mounted on the latter.

A pliable aluminum rod with a dim red LED at its end was attached to the helmet at a distance of about 0.40 m in front of the listener’s eyes, such that it was approximately aligned with the center LED of the hemisphere with the head in the comfortable, straight-ahead orientation. Listeners were instructed to use this rod-LED as a pointer to indicate the perceived sound direction. In this way, it was guaranteed that listeners always pointed their head with the eyes in a fixed, roughly straight-ahead, orientation in the head. A firm neck rest allowed for a reproducible and stable initial orientation of the listener’s head at the start of each trial throughout the session.

### Experimental paradigm

The coordinates of target locations and the endpoints of the localization responses were all described in a double-pole azimuth-elevation coordinate system, in which the origin coincides with the center of the head [21]. The azimuth angle, *α*, is defined as the angle within the horizontal plane relative to the vertical midsaggital plane, whereas the elevation angle, *ε*, is defined as the direction within a vertical plane relative to the horizontal plane through the listener’s ears. The relation between the [*α*, *ε*] coordinates and the polar [*E*, Φ] coordinates defined by the LED hemisphere (see above) is given in [10].

Each experimental session started with a calibration run in which the listener had to align the rod LED with 36 peripheral LEDs on the hemisphere, presented in random order. Subsequently, the experiments with the rippled noise stimuli were performed. Stimuli with different ripple bandwidths were presented in separate recording sessions. All stimuli were presented with the speaker at the straight-ahead position ([*α*, *ε*] = [0, 0]°).

An experimental session consisted of two runs in which each of the 175 stimulus spectra were presented once in randomized order. Each stimulus spectrum was thus presented twice in one session. A trial always started with an initial fixation light at *α* = -14° or +14° azimuth (randomly selected), and at 0° elevation. Then, after a randomized period between 0.9 and 1.1 seconds, the fixation LED was switched off and the sound was presented. Head position was measured for 3.0 sec after onset of the fixation spot. listeners were instructed to reorient their head as fast and as accurately as possible in the apparent sound direction. Although listeners were aware of the fixed speaker at the straight-ahead location, they were encouraged to respond to the perceived apparent sound direction, rather than to the (remembered) actual speaker location.

All listeners participated at least once in all three sessions to the stimuli with different ripple bandwidths. Listener S5 performed in two sessions with the 3.0 c/o-bandwidth stimuli. Listener S3 participated in three sessions with the 3.0 c/o-bandwidth stimuli, and in two sessions for both the stimuli with a 1.5 and a 5.0 c/o bandwidth.

Listener S2 participated in four additional sessions in which the speaker was positioned at [*α*, *ε*] = [0, 80]°, i.e. above the listener. For these experiments, only the stimuli with the ripple bandwidth of 3.0 c/o were used. In this condition, the listener perceived some of the stimuli in the rear hemisphere. In that case, he was instructed to point in the direction of the perceived sound location, mirrored with respect to the frontal plane (i.e. in the frontal hemifield). For example, if he perceived the sound to be at an elevation of 120 deg (i.e., 30 deg beyond the zenith), he should point to a location at 60 deg upward relative to straight ahead. This pointing task thus prevented the subject from having to make a mechanically nearly impossible motor response to the perceived target. To identify off-line in which trials the listener perceived stimuli at the rear, he was also instructed to press a button as soon as the pointing was completed.

### Data analysis

The raw head-position signals (measured in Volt) and the corresponding LED coordinates (in degrees) from the visual calibration run were used to train two three-layer backpropagation neural networks that mapped the raw head-data signals when pointing at the LEDs into calibrated head position signals in azimuth-elevation angles [10]. Each network had two input units (horizontal and vertical field measurements, respectively), four hidden units, and one output unit (either azimuth, or elevation angle). The networks corrected for small inhomogeneities of the magnetic fields, and slight crosstalk between the horizontal and vertical channels that resulted from small deviations from perfect orthogonality of the fields, and typically yielded an absolute accuracy of one degree, or better, for targets across the frontal hemifield.

A custom-made Matlab script [10] was used to identify saccades in the calibrated head-position signals on the basis of preset velocity criteria for saccade onset and offset, respectively. The endpoint of the first saccade after stimulus onset was defined as the response position. All saccades were visually checked and corrected if necessary. Saccades with latencies shorter than 80 ms or longer than 800 ms were discarded from further analysis. Earlier responses are usually predictive, whereas later responses are considered to be caused by inattention. In the case of rear responses, the signal was immediately reset to zero at the moment the listener pushed the button. The occurrence of such resets was labeled to indicate a percept in the rear hemifield. For these trials, the perceived elevation in the rear hemisphere, *ε*_*r*_, was calculated by adding 180° to the measured frontal elevation, *ε*_*f*_ (see above).

### Measurement of Head-Related Transfer Functions (HRTFs)

Head related transfer functions were measured for all listeners for 25 different elevations (*ϵ* = -60°, -55°, …, 55°, 60°) and at a fixed azimuth, *α* = 0°. A periodic flat-spectrum Schroeder-phase signal (FM-sweep-like signal, [22]) was used as a stimulus. It consisted of 20 periods of 20.5 ms, which adds up to a total stimulus duration of 410 ms. The spectrum was flat within 0.2–20 kHz, and the sound level at the listener’s head was 65–70 dB SPL.

Pressure waveforms near the entrance of the ear canal were measured with a miniature microphone (Knowles EA1842) attached to a thin tube (1.5 mm diameter). The tube was kept in place by a thin ring attached to a custom-made thin metal rod that was positioned to the side of the head with a head band. The listener was seated in a chair in the center of the experiment room.

The microphone signal was amplified by a measurement amplifier (Bruël & Kjær 2610), subsequently fed into a bandpass filter (Krohn Hite 3343, passband 0.2–20 kHz), and finally sampled at 50 kHz by a data acquisition board (Data Translation DT3818). From period 2–19 of the sampled (periodic) waveforms, the average signal per period was computed (containing 1024 samples), and transformed into 512 spectral bins (resolution 48.8 Hz) by means of the Fast Fourier Transform. The directional transfer functions (DTFs) were then computed by subtracting the mean amplitude spectrum (in decibels) computed over the entire set.

### Reconstruction of the elevation-related spectral shapes

The data from the localization experiments are used to reconstruct elevation-specific spectral shapes, by applying the method described in [19]. In short, smooth response distributions to a given rippled stimulus, *X*_*k*_(*ω*), here denoted by *p*(*ε*|*X*_*k*_(*ω*)), with *k* ∈ 1⋯175, were constructed by replacing each elevation response to that stimulus, *ε*_*ik*_, by a normalized Gaussian, centered at the response elevation, with a width in the elevation direction of *σ*_*ε*_ = 2 deg, and by summing all Gaussians (typically, *i* ∈ [1, 2], see e.g. Fig 6). Thus, *p*(*ε*|*X*_*k*_(*ω*)) can be interpreted as the probability of a response to elevation *ε*, when stimulus *X*_*k*_(*ω*) is presented (the so-called likelihood function in Bayesian estimation). Note, that it is important that the resulting probability distributions are unimodal, indicating that there is a unique stimulus-response relationship (see e.g. Figs 5 and 6).

**Fig 3:**
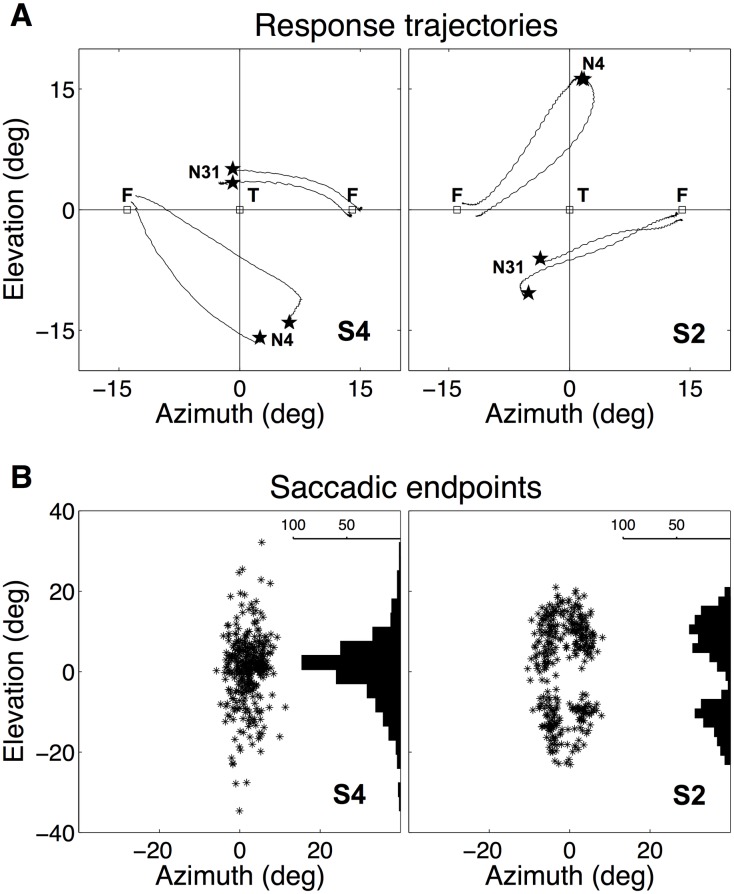
Example responses. **A:** Response trajectories of head movements of listeners S4 (left) and S2 (right) to two stimuli (stimulus numbers N4 and N31). F indicates the fixation LEDs and T the speaker position. Note that listeners perceived the stimuli at different elevations, and that their responses were consistent. **B:** Response scatter to all stimuli for listeners S4 and S2, together with the elevation response distribution. Data for the spectra with a ripple bandwidth of 3 c/o.

In the present study, we aimed to estimate the spectral features that underlie the percept of elevation angle *ε*, which we here denote by *P*(*ω*; *ε*). As a first step toward this estimate, we need to determine the probability that stimulus *X*_*k*_(*ω*) was presented, given a particular response elevation, *ε*. This conditional probability, described by *p*(*X*_*k*_(*ω*)|*ε*), can be computed from Bayes’ rule, according to:

$$p\left(X_{k}\left(\omega \right)|\varepsilon \right)=\frac{p\left(\varepsilon |X_{k}\left(\omega \right)\right)\cdot p\left(X_{k}\left(\omega \right)\right)}{p(\varepsilon )}$$ (3)

in which *p*(*X*_*k*_(*ω*)) is the (expected) unconditional probability of spectral shape *X*_*k*_(*ω*), known as the *prior* distribution. The normalization factor, *p*(*ε*), represents the total distribution of elevation responses irrespective of the stimulus spectrum, and equals the normalized distribution of all elevation responses (see e.g. Fig 3B). Note, that in Eq 3 both the latter distribution, and the conditional probability *p*(*ε*|*X*_*k*_(*ω*)) can be extracted from the experimental data. However, the prior, *p*(*X*_*k*_(*ω*)), is in principle unknown, as it may be partly determined by (idiosyncratic) expectations about stimulus spectra, by previous experience, or by other covert factors that could not be controlled in the experiment. To circumvent the problem of having to estimate the prior distribution, we therefore took a more pragmatic approach by determining the unconditional probability of stimulus *X*_*k*_(*ω*) from the actual distribution of applied stimulus spectra. This distribution was taken to be *uniform* in our experiments, as the occurrence of each particular stimulus was equally likely, and completely randomized. Thus, *p*(*X*_*k*_) ≡ 1/175, for all *k*, and as a consequence, Bayes’ rule becomes a maximum likelihood estimate (MLE):

$$p\left(X_{k}\left(\omega \right)|\varepsilon \right)=\frac{p\left(\varepsilon |X_{k}\left(\omega \right)\right)\cdot p\left(X_{k}\left(\omega \right)\right)}{\sum_{n=1}^{175}p\left(\varepsilon |X_{n}\left(\omega \right)\right)\cdot p\left(X_{n}\left(\omega \right)\right)}=\frac{p(\varepsilon |X_{k}\left(\omega \right))}{\sum_{n=1}^{175}p\left(\varepsilon |X_{n}\left(\omega \right)\right)}$$ (4)

If the MLE for *p*(*X*_*k*_(*ω*)|*ε*) is large, more responses to elevation *ε* are made for stimulus spectrum *X*_*k*_(*ω*) than for other spectra, and thus stimulus *X*_*k*_(*ω*) will contain spectral features that contribute significantly to the perceived elevation angle *ε*.

As will become apparent from the data, a given elevation is typically perceived for a number of rippled spectral shapes. This suggests that the different spectra may either contain a common spectral feature that is responsible for the percept (like expected from the CPA model, or the spectral derivative model), or a number of different spectral features that all contribute to the same perceived elevation angle. Thus, to estimate the actual spectral features underlying the perceived elevation angle, *P*(*ω*; *ε*_*p*_), all rippled spectra that gave rise to that percept should somehow be incorporated. If, for example, *ε*_*p*_ would be determined by a single spectral feature, say, a CPA or a notch (see Introduction), it should emerge as the common feature in all contributing rippled spectra. Following the procedure of [19], we thus adopted a linear weighting scheme to estimate *P*(*ω*; *ε*_*p*_).

From the response-dependent MLEs, *p*(*X*_*k*_(*ω*)|*ϵ*), extracted from the data for each rippled spectrum through Eq 4, we estimated the elevation-dependent spectral features giving rise to a perceived elevation angle, *ϵ*_*p*_, by taking a weighted sum of the rippled stimulus spectra *X*_*k*_(*ω*) that contributed to this perceived elevation, and let the MLEs act as linear weights [19]:

$$P\left(\omega ;\varepsilon_{p}\right)=\sum_{k=1}^{N_{\varepsilon_{p}}}\;p\left(X_{k}\left(\omega \right)|\varepsilon_{p}\right)\cdot X_{k}\left(\omega \right)$$ (5)

with *N*_*ε*_*p*__ the number of stimuli contributing to percept *ε*_*p*_.

Note, that in the actual experiments, the sensory spectrum was not equal to the free-field rippled spectrum *X*_*k*_(*ω*), because it is determined by the convolution between the free-field stimulus spectrum and the HRTF associated with the straight-ahead speaker location at *ε*_0_ = 0 deg, *H*(*ω*; *ε*_0_) (Eq 1). Thus, the sensory spectrum for stimulus *k* should be estimated by:

$$S_{k}\left(\omega ;\varepsilon_{0}\right)=H\left(\omega ;\varepsilon_{0}\right)\cdot X_{k}\left(\omega \right)$$

In the Hofman and Van Opstal [19] study, this aspect was not incorporated in the analysis. In the present paper, we have accounted for this difference to estimate the perceptual spectral features from more faithful representations of the sensory inputs. Thus, Eq 5 is modified to:

$$P_{0}\left(\omega ;\varepsilon_{p}\right)=\sum_{k=1}^{N_{\varepsilon_{p}}}\;p\left(S_{k}\left(\omega ;\varepsilon_{0}\right)|\varepsilon_{p}\right)\cdot S_{k}\left(\omega ;\varepsilon_{0}\right)$$ (6)

in which the subscript *P*_0_(⋅) indicates that the free-field stimuli have been converted into sensory spectra.

In our reconstruction algorithm, we implicitly assumed that the localization response depends only on the sensory spectrum, and is not influenced by other non-acoustic factors, such as head orientation, or expectation. Moreover, we assumed, for simplicity, that in case a stimulus would contain spectral shape features relating to multiple elevations, that the listener’s response was not determined by averaging over the different elevation angles (see, e.g., [17]).

### Spectral correlation

Quantitative comparisons between the profiles of two magnitude spectra, *A*(*ω*) and *B*(*ω*), were performed by computing the spectral correlation coefficient, *C*_*AB*_ [8]:

$$C_{AB}\equiv \frac{\left\langle A(\omega )B(\omega )\right\rangle }{\sqrt{\left\langle A^{2}\left(\omega \right)\right\rangle -{\left\langle A(\omega )\right\rangle }^{2}}\sqrt{\left\langle B^{2}\left(\omega \right)\right\rangle -{\left\langle B(\omega )\right\rangle }^{2}}}$$ (7)

with the spectral mean of *X*(*ω*) defined as

$$\left\langle X\;(\omega )\right\rangle \equiv \frac{1}{\omega_{2}-\omega_{1}}\;\int_{\omega_{1}}^{\omega_{2}}\;d\omega X\;\left(\omega \right)$$ (8)

We took *ω*_1_ = 4 kHz and *ω*_2_ = 14 kHz. The amplitude spectrum, *A*(*ω*) or *B*(*ω*), was specified in decibels, and frequency, *ω*, was given in octaves. One can interpret *C*_*AB*_ as a similarity index that lies in the range [-1, +1]. Maximum similarity corresponds to *C*_*AB*_ = +1, and occurs when *A*(*ω*) can be expressed as *A*(*ω*) = *pB*(*ω*) + *q* (with *p* and *q* constants). No similarity occurs when the *C*_*AB*_ index is close to zero, or becomes negative.

## Results

Although the speaker was always positioned at [*α*, *ε*] = [0, 0]°, and listeners were aware of this fact, head-movement responses were nevertheless distributed over a considerable range of elevations for most listeners. Note, that prior knowledge about the speaker position is in fact a disadvantage for these experiments, as the stimuli often do not appear to come from that direction, causing potential confusion. Listeners were thus encouraged to indicate the actually perceived direction of the sound, and to disregard the (remembered) physical speaker position. However, this task did not cause listeners to make random localization movements away from [*α*, *ϵ*] = [0, 0]°, as the responses to the two stimulus presentations in the two runs within one session, and also between sessions taken on different days, were very consistent. As an example, Fig 3A shows typical responses to the repeated presentation of two different rippled stimuli, each with a ripple bandwidth of 3 c/o (stimulus numbers N4 and N31) for two listeners, S4 and S2. Note that the listeners respond to different elevations for these stimuli; S4 localizes N31 around 5 degrees upward from the straight ahead center location, and N4 around 15 degrees downward, whereas S2 localizes N31 around 7 degrees downward, and N4 around 15 degrees upward.

Note also the reproducibility of the listeners’ responses, as the head movement trajectories end at the same location. When Pearson’s correlation is computed between the elevation components of the first and second response to the same stimulus, the data from both listeners yielded high correlations: *r* = 0.82 for S4, and *r* = 0.86 for S2 (see below, Fig 5, and Table 2).

However, when the correlation coefficient was computed between the responses of S4 and S2 for the same stimuli, it was much lower (*r* = 0.35), which indicates that these listeners typically perceived the same stimulus at quite different locations.

Fig 3B shows the distributions of the response data for the 3 c/o stimuli of both listeners. Both had a response range that extended from about -25° to +25° in elevation. In azimuth, the response endpoints remained close to the midline at *α* = 0°. Whereas the majority of the responses of listener S4 clustered around the veridical speaker location at 0° elevation, the responses of participant S2 were mainly directed away from this location, leading to a bimodal distribution of elevation responses clustered around a slightly upward (about 5 deg) and a slightly downward (-5 deg) elevation. This bimodal response pattern perhaps betrays a strategy of this listener to actively avoid the (known) physical location of the sound source at elevation zero, and a reluctance to move to that location. Notwithstanding such a potential bias, the responses to the different noise spectra were highly consistent and reproducible, also for this subject. This reproducibility ensured that the major component in the saccadic responses of our listeners were driven by an *acoustic, spectral* evaluation of the sensory input, despite the fact that all were aware of the frequent substantial mismatches between actual and perceived sound-source locations (see Discussion).

**Fig 4:**
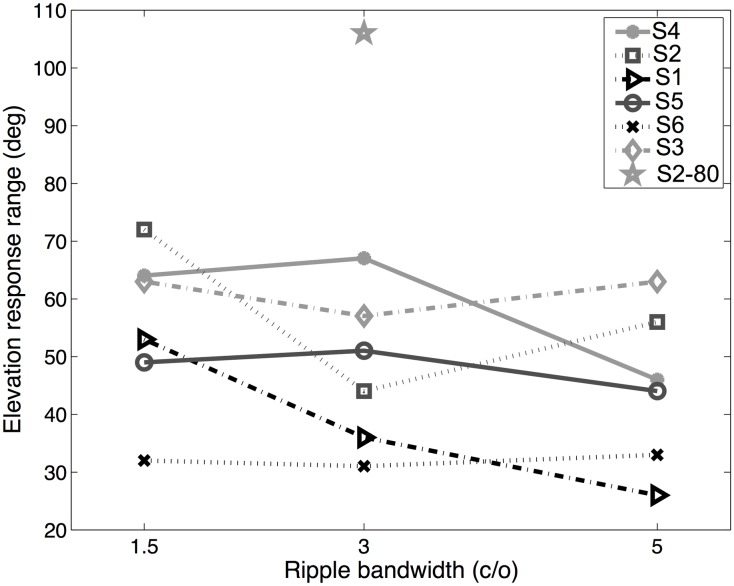
Response ranges. Elevation response range as a function of ripple bandwidth for all listeners. The star at 3 c/o indicates the data of S2 with the speaker at [*α*, *ϵ*] = [0, 80]°. There is no systematic relation between stimulus bandwidth and response range.

Table 1 provides the medians and standard deviations for the elevation responses of all listeners and all three stimulus sets. For listeners S1, S4, and S6 the responses were distributed around 0° elevation, whereas the responses of listeners S3 and S5 were distributed around slightly upward elevation angles. More importantly, however, is the observation that there is no systematic difference between the response distributions for the three stimulus sets: the mean value, standard deviation, and response range are independent of the ripple bandwidth for each of the listeners. This important point is illustrated in Fig 4, which shows the response range for all subjects, as function of the ripple bandwidth. No systematic relation emerged, indicating that the response distributions were insensitive to the variations in the amplitude spectra.

**Table 1:** Medians and standard deviations (in degrees) of the response distributions for the three stimulus sets with different ripple bandwidths for all listeners.

Bandwidth	S1	S2	S3	S4	S5	S6	S2-80
1.5 c/o	0.75 (4.22)	8.82 (14.37)	6.10 (10.40)	-0.07 (8.24)	21.10 (5.24)	1.82 (4.73)	
3.0 c/o	0.59 (3.56)	5.09 (11.59)	13.23 (10.86)	1.16 (7.77)	15.05 (5.48)	2.77 (3.35)	27.22 (24.09)
5.0 c/o	3.95 (1.97)	8.97 (12.27)	5.58 (8.01)	1.13 (6.07)	20.08 (8.30)	2.54 (4.10)	

**Fig 5:**
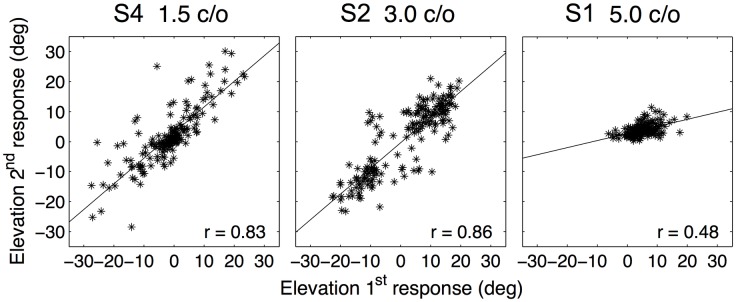
Response consistency. Elevation responses of the second run vs. elevation responses of the first run together with a regression line and the correlation between the two runs (indicated in the lower right corner). Data of listener S4 for the 1.5 c/o stimuli, listener S2 for the 3.0 c/o stimuli and listener S1 for the 5.0 c/o stimuli. Both S4 and S2 responded over a considerable range and show a high correlation between the two presentations of one stimulus. Listener S1 responded over a more restricted range, and the correlation between the two runs was lower, although still highly significant.

Not only did listeners respond over a considerable elevation range, their responses to the two presentations of each stimulus were also quite consistent. Fig 5 shows, for three of the listeners, the two response elevations for each stimulus plotted against each other. Listeners S2 and S4 responded over a larger elevation range than listener S1, and their correlations were also higher (*r* = 0.83, 0.86, and 0.48, respectively). The correlations between the responses of the two runs are given in Table 2 for all listeners and stimulus sets. Correlations were usually high and always significant. Listeners S2 and S4, who were the most experienced subjects in open-loop sound-localization experimental procedures, yielded correlations between 0.82 and 0.92, but also listener S6, who was inexperienced in these type of experiments, showed high correlations. For listeners S3 and S5, who performed more sessions for one or all of the stimulus sets, we also calculated the correlation between the diferent sessions. For listener S5 the correlation between the two sessions of the 3 c/o stimuli was 0.61. For listener S3 the correlations between the different sessions of one stimulus set varied between 0.50 and 0.61. These values are in the same range as the correlations between two runs of one session, which shows that even in different sessions, on different days, listeners consistently assigned a particular elevation to a certain stimulus.

**Table 2:** Correlations between the elevation components of the two responses toward each stimulus for all three stimulus sets and all listeners.

Bandwidth	S1	S2	S3	S4	S5	S6	S2-80
1.5 c/o	0.57	0.87	0.63, 0.73	0.83	0.73	0.83	
3.0 c/o	0.64	0.86	0.61, 0.54, 0.55	0.82	0.72, 0.62	0.74	0.89, 0.90, 0.87, 0.84
5.0 c/o	0.48	0.92	0.70, 0.58	0.82	0.53	0.68	

**Fig 6:**
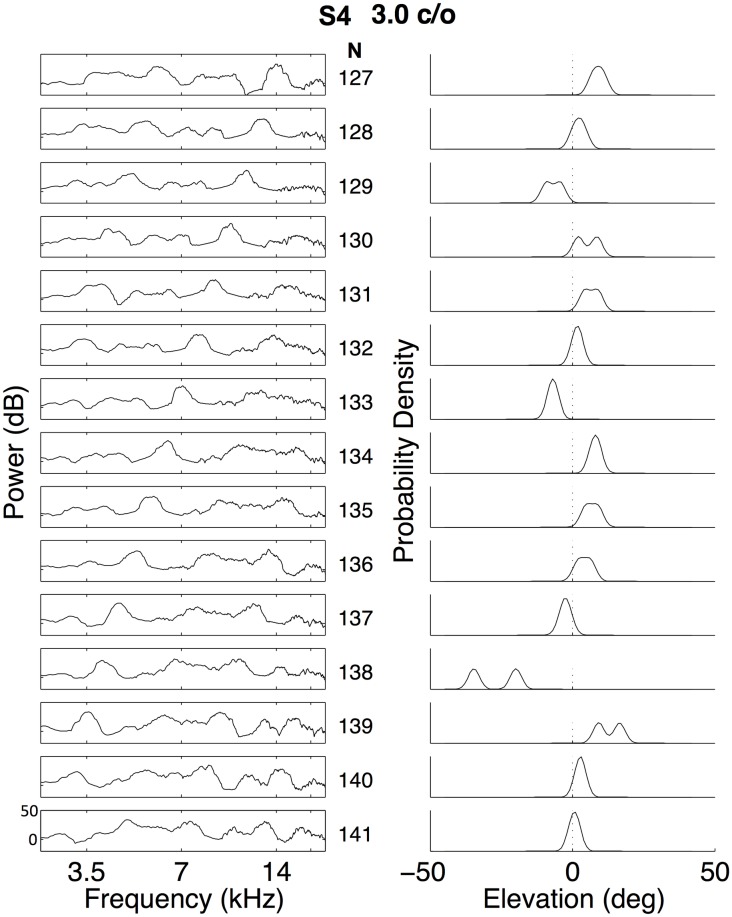
Single-stimulus response distributions. Subsequent stimulus spectra (left, stimulus number indicated on the right) with their probability density distributions for listener S4. Stimuli were presented in random order. Note that certain stimulus sequences, e.g. N = 127–129, 130–133, 134–138, and 139–141 yield a systematic shift in the perceived elevation. Note also, that a given elevation, e.g. 5 deg upward, is perceived by different stimulus spectra (here: stimulus numbers, N = 127, 131, 134, and 139).

An important requirement for the reconstruction procedure is that the conditional response probabilities, *p*(*ε*|*X*_*k*_(*ω*)), are described by (near-)unimodal distributions, indicating that a given stimulus yielded a unique elevation percept. As described in the Methods, each elevation endpoint was replaced by a normalized Gaussian (width 2 deg). Response Gaussians for the same stimulus were subsequently added to estimate the elevation response distribution for a given stimulus. To illustrate this procedure, Fig 6 shows fifteen consecutive stimulus spectra and their corresponding response distributions for listener S4 for the 3 c/o stimuli. In line with the high correlation observed in Fig 5, the majority of response distributions were indeed unimodal. As explained in the Methods, in stimulus spectra with subsequent ranks, the window defining the filter shape to create the stimulus was shifted by 1/6 octave across the rippled root sequence. As a result, spectral features shift to lower frequencies by 1/6 octave for consecutive rippled spectra. This aspect of the stimuli is apparent in the left panel of Fig 6. For example, the peak at 14 kHz in stimulus number N127 is located at 7 kHz in N133, and at 3.5 kHz in N139. Note, that as the spectral features move from higher to lower frequencies, the response distributions tend to shift in elevation in a systematic way.

**Fig 7:**
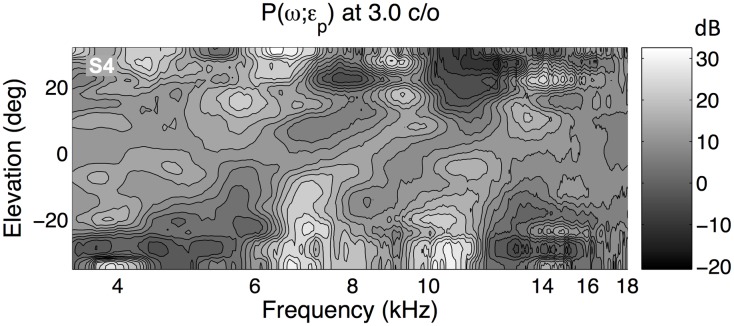
Bayesian spectral reconstruction. Reconstructed spectral features for the 3 c/o stimuli for listener S4, based on the free-field stimulus spectra (Eq 5). Abscissa indicates perceived elevation angle (in deg), ordinate frequency (log scale). Amplitude (in dB) is encoded in grayscale, where light shades indicate peaks, and dark shades indicate notches in the spectrum.

Conditional response distribution, as are shown in Fig 6, together with the normalized unconditional distributions of elevation responses as shown in the histograms of Fig 3B, were then used to compute the MLE for each stimulus, given the response elevation, *p*(*X*_*k*_(*ω*)|*ε*), by applying Eq 4. These MLE estimates were subsequently used as linear weights for each of the corresponding stimulus spectra to construct the elevation-dependent spectral shapes underlying the percept of a given elevation angle, *P*(*ω*; *ε*_*p*_), with Eq 5. In Fig 7 we show the reconstructed perceptual spectral features for listener S4 over the 3–18 kHz band for sounds with a ripple bandwidth of 3 c/o, in the same format as in the study of Hofman and Van Opstal [9]. The abscissa represents the listener’s perceived elevation, *ε*_*p*_, while the amplitude of the spectra (in dB) is encoded in gray scale: light shades correspond to a peak in the spectrum, while dark shades indicate a spectral notch. Note that the reconstructed perceptual spectral features have a rich structure. Rather than a single peak or notch, a complex combination of peaks and notches appears to underly the listener’s perceived elevation direction. Thus, this result does not support models that explain the extraction of elevation on the basis of a CPA, or a single notch (see Introduction).

**Fig 8:**
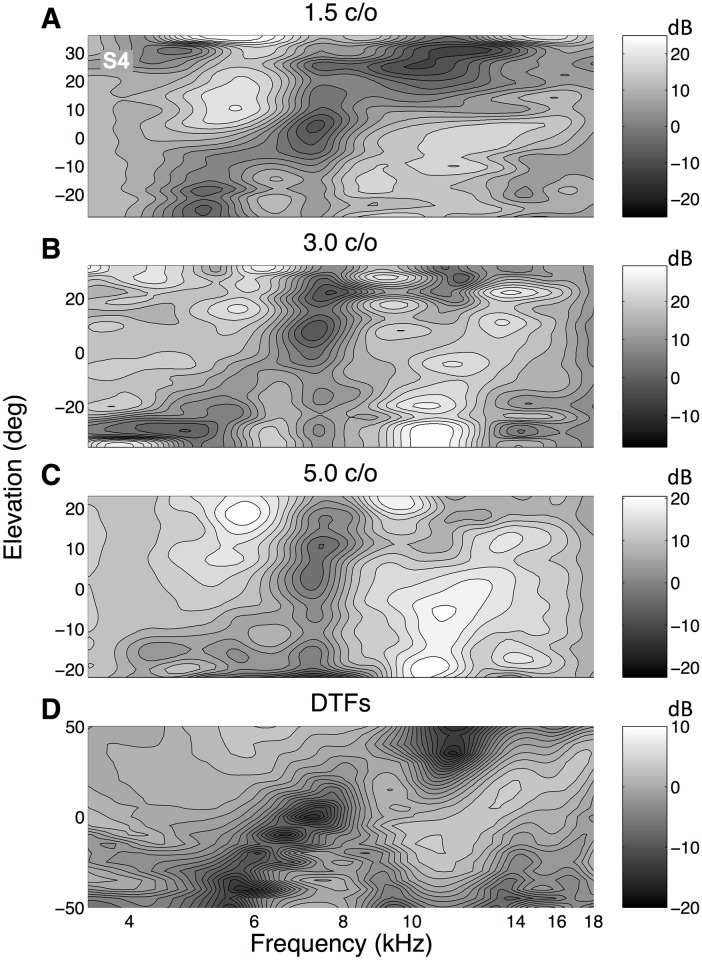
Different bandwidths. Reconstructed spectral features for the three different amplitude-spectral bandwidths, based on the sensory spectra, together with the DTF for listener S4. Same format as Fig 7. Note that the three reconstructed spectra appear to be very similar, despite the considerable differences in the underlying stimulus spectra (e.g. Fig 2). Note also the remarkable similarities in the reconstructed patterns with the DTFs of this listener. For example, the diagonal notch from about 5 to 8 kHz, and the peak running from around 10 to 15 kHz, can be seen both in the reconstructed spectra and in the DTFs.

The reconstructed perceptual spectral shapes in Fig 7 are based on the free-field stimulus spectra, *X*_*k*_(*ω*), rather than on the sensory spectra, *S*_*k*_(*ω*), delivered to the eardrum. As outlined in the Introduction and the Methods, the latter are more relevant to the auditory system. To estimate the sensory spectrum, the contribution of the DTF for the straight-ahead speaker location was added to the free-field spectrum (with weight, *γ* = 1), and the corrected perceptual features were then reconstructed by applying Eq 6. The results are shown in Fig 8 for the three ripple bandwidths for listener S4, together with the listeners’ DTFs for comparison (bottom panel). Note the changes in the perceptual spectral shapes, when compared to the uncorrected data of Fig 7. More importantly, however, the three reconstructed spectra resulted to be remarkably similar, not only for the different ripple bandwidths, but also to the listener’s DTFs. Prominent features in the DTFs, like the notch running from about 5 kHz for downward elevations, to about 8 kHz for upward elevation angles, are clearly visible in the three reconstructed perceptual spectral shapes. But also the diagonal peak from about 10 kHz to 15 kHz, the secondary notch around 10 kHz, the peak around 5 kHz for upward elevations, and the peak near 7 kHz for downward locations were found in the different reconstructions. This result is quite remarkable, considering that the entire reconstruction is based on only two (open-loop) head-movement responses per stimulus, that the stimulus sets themselves were highly dissimilar (e.g. Fig 2), and the underlying model is extremely simple (linear weighting of stimulus spectra).

**Fig 9:**
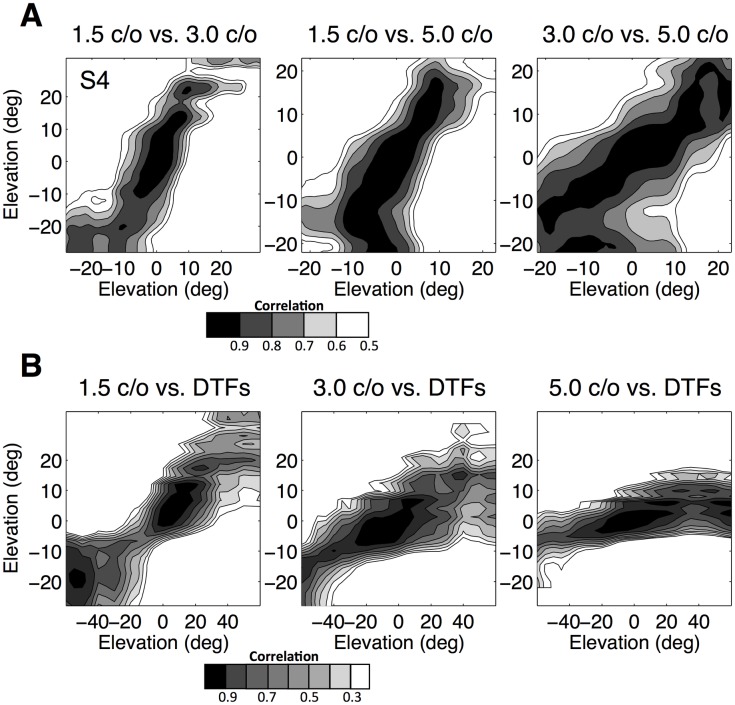
Spectral correlations. **A:** A measure of the similarity between the different reconstructed spectral shapes of listener S4 is given by the correlation matrix, *C*(*ε*_1_, *ε*_2_). Grayscale values indicate correlation values between 0.5 and 1, with the contour lines at 0.1 steps, with darker grayscale values for higher correlations. **B:** The correlation matrix for the three reconstructed spectra and the DTFs of listener S4. Grayscale values indicate correlation values between 0.2 and 1. The correlation matrix is high only for locations on, or near, the main diagonal.

To quantify the similarity between the different reconstructed patterns, Fig 9A shows the spectral correlations between the three reconstructed spectra of listener S4 (see Methods). Grayscale values indicate correlations between 0.5 to 1, with darker gray shades corresponding to higher correlations. Fig 9B shows the correlations between the three reconstruced spectral shapes and the DTFs of listener S4, with grayscale values indicating correlations between 0.2 and 1.0. To construct these latter plots, the reconstructed spectra had to be resampled to enable the comparison. For both comparisons the correlations are highest around the diagonal and decrease for elevations away from the diagonal, although the results for the 5 c/o stimuli were more variable.

Note that despite the remarkable similarities, an important difference between the reconstructed spectral shapes and the measured DTFs was found in the range of the elevation angles (roughly, between -20 and +20 deg for the perceived elevations, vs. -60 to +60 deg for the DTFs, see Methods). Clearly, the prominent spectral shape of the DTF corresponding to the straight-ahead speaker location was unavoidably added to the random spectral shapes of the free-field stimuli, and therefore the listener’s elevation percept was likely to be influenced by the presence of this DTF in the sensory spectrum. As explained in the Methods, the reconstruction assumes that the listener’s percept was determined by a unique elevation angle, and was not designed to cope with the possibility that the system may actually average across different potential elevation angles to determine the perceived elevation. To illustrate the influence of the speaker-location induced DTF on the sensory spectrum, we conducted a series of four experiments with the 3.0 c/o stimuli with listener S2, in which we positioned the speaker at [*α*, *ε*] = [0, 80]° (again, the listener was aware of the actual speaker location). The DTFs corresponding to far-upward directions are considerably flatter than for frontal directions (the spatial resolution in upper space is worse too [23]), and therefore we expected the speaker at 80 deg to dominate the sensory spectrum to a lesser extent.

**Fig 10:**
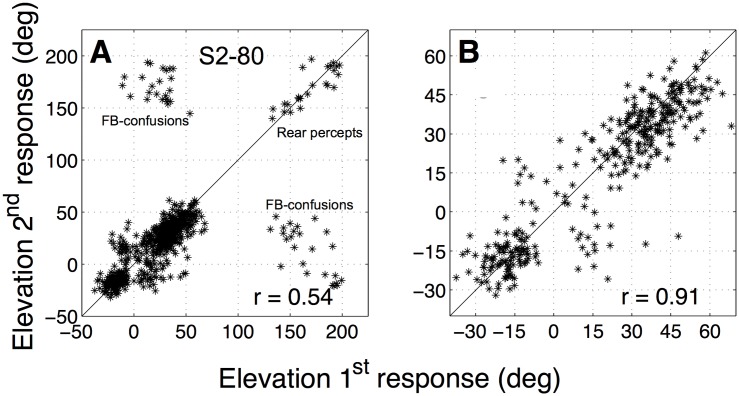
Overhead speaker location. Elevation responses of the second run vs. elevation responses of the first run for the experiment with the speaker at (0,80)° for listener S2 (pooled data for all four sessions). Correlations between the two runs are indicated in the lower right corner. Data Panel A shows the data including the responses perceived in the rear hemisphere (*ε*_*p*_ > 90°). The correlation is low, mainly because of front-back confusions (points far off-diagonal). In panel B, all responses to the rear and all front-back confusions were omitted, leading to a substantially higher response correlation over a larger elevation range than for the straight-ahead speaker (cf. Fig 5B).

Indeed, in these experiments the listener’s elevation responses covered a much larger range. Fig 10A shows the responses of the second run versus the responses of the first run for all sessions. Although most responses were in the frontal hemisphere (*ε*_*p*_ ∈ [−90, +90]°), front-back confusions and reversals occurred too. Data points with *ε*_1,2_ > 90° indicate stimuli for which the sound was consistently perceived at a rear location (a true rear percept). A front-back confusion occurred when data points in Fig 10A ended in either the upper left, or the lower right corner of this plot. Note, however, that such front-back confusions were not random; they tended to cluster around the diagonal with a slope of minus one, indicating that the participant had a clear elevation percept, but only front vs. back could be ambiguous. In Fig 10B we show the correlation plot when all rear and confused responses were not included. The correlations of the data (here: *r* = 0.91) were then very similar as for the experiment with the speaker at [*α*, *ε*] = [0, 0]° (*r* = 0.86; see Table 2), but distributed over a much larger elevation range.

**Fig 11:**
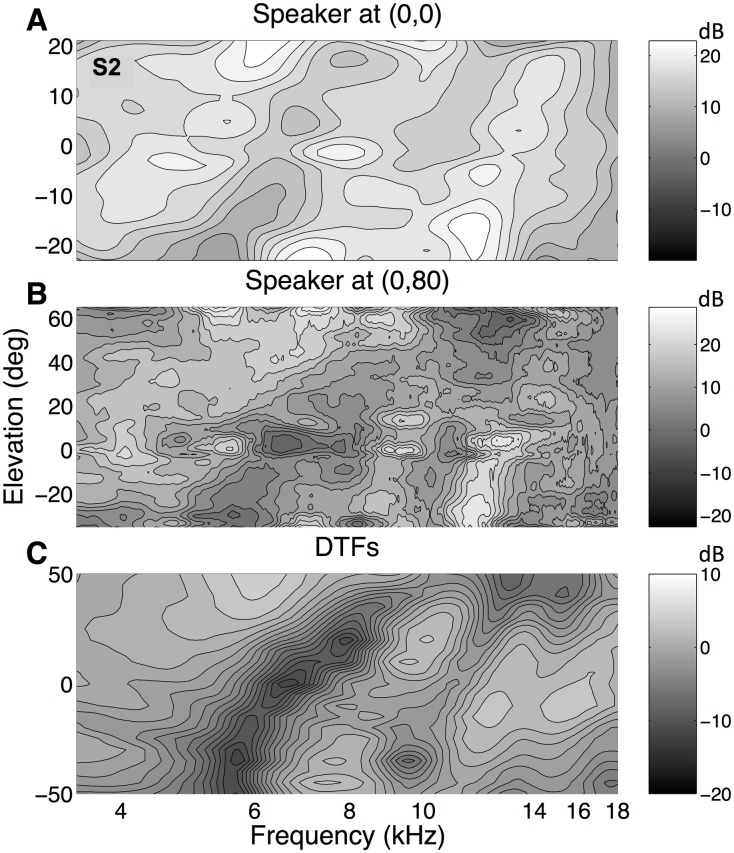
Spectral reconstructions. Reconstruction of the spectral features for listener S2, based on the elevation responses to the 3 c/o stimuli from the frontal speaker location (panel A; corrected for the straight-ahead DTF, weight 0.5) and from the overhead speaker location (panel B; only responses into the frontal hemifield; not corrected for the speaker’s DTF). Panel C shows the measured DTFs of listener S2. Again, there is a high similarity between the two reconstructed spectra, and with the listener’s DTFs. Note the differences in scale of the elevation axes in the different panels. Same format as Fig 7.

From these frontal responses we again reconstructed the perceptual spectral shape functions. The results are shown in Fig 11, which compares the spectra for the two speaker positions (straight ahead, Fig 11A, corrected for the sensory spectrum, here *γ* = 0.5; upward: Fig 11B, uncorrected) with the listener’s DTFs (Fig 11C). The same spectral features can be seen in the two reconstructed spectra and in the listener’s DTFs, like a notch running from 5 kHz for low elevations to about 8 kHz for high elevations, and a peak from 11 kHz to 14 kHz. However, the perceived elevation range increased from [−20, +20]° to about [−30, +60]° by moving the speaker to [*α*, *ε*] = [0, 80]°.

## Discussion

In this paper, we showed that random broadband spectra with different degrees of spectral variation are systematically mislocalized. Even though the speaker was always located at [*α*, *ϵ*] = [0, 0]°, and listeners were aware of this fact, they consistently responsed over a broad range of elevations. Note that in the stimulus generation we did not compensate for the HRTF of the speaker position in the original stimulus spectrum, so that the sensory spectrum at the eardrum was likely to be dominated to a great extent by the HRTF at 0° (Eq 6). This probably explains why many responses were clustered around 0° elevation for most listeners. Although only tested for one of the listeners, the elevation response range increased drastically when the speaker was positioned at [*α*, *ϵ*] = [0, 80]° instead of at [0, 0]°. The HRTF at 80° is much flatter than the HRTF of straight ahead (and, consequently, the spatial resolution around the zenith is relatively poor, [23]), and is therefore expected to have less influence on the shape of the sensory spectrum than the HRTF for straight-ahead. Yet, all listeners responded quite consistently to different presentations of a given spectral shape, even on different days, indicating that listeners consistently assigned a particular elevation to a certain stimulus spectrum.

Remarkably, we obtained no front-back confusions in the experiment with the speaker positioned at [*α*, *ϵ*] = [0, 0]°, despite the full randomness of spectral shapes in our stimulus sets. Although it is not entirely clear which particular spectral cues are crucial to resolve or induce front-back confusions (e.g., [24]), the spectral imprint of the straight-ahead speaker probably dominated any potential front-back confusion by the random spectra. However, when the speaker was moved to 80° elevation, for which the HRTF has a much flatter shape, the random spectral shapes could now dominate the overall sensory spectrum, leading to a substantial increase in the number of front-back confusions (Fig 10).

On the basis of the sensory spectra and the response distributions, we reconstructed elevation-dependent spectral shapes for all listeners and all three stimulus sets. For all listeners, the reconstructed spectra for the three stimulus sets were very similar (Fig 9). Moreover, distinctive features of the HRTF of a particular listener can also be found in the reconstructed spectra, although the elevation range was more restricted in the latter. A plausible explanation for the restricted elevation-response range lies in the dominance of the straight-ahead HRTF in the sensory spectrum (Eq 6), causing the auditory system to average between the straight-ahead elevation and the spectral features pointing to alternative elevation angles. The existence of an averaging mechanism for sounds in the midsaggital plane has been demonstrated convincingly in synchronous double-sound experiments [17].

### Effect of bandwidth

Broadband sound stimuli with flat spectra, emanating from a single location, are typically localized accurately ([2], [10], [15], [25], [26]), with a spatial resolution of about 4–5 deg in the vertical plane. Similar accurate performance would be expected for broadband stimuli for which the spectral variations are too fast to be perceived by the human auditory system. It is not well known how localization accuracy is affected by the spectral variations in between these two extremes. Kulkarni and Colburn [16] presented listeners with sounds filtered with HRTFs with several degrees of spectral detail, and with ‘real’ sounds. HRTFs could be smoothed significantly in the frequency domain before affecting the perceived location of the sound stimulus. Hofman and Van Opstal [19] reported that random, broadband spectra with a ripple bandwidth of 3 c/o, are consistently mislocalized in elevation. Note that a spectral variation of 3 c/o roughly corresponds to one cycle per critical band.

In the present experiments we used rippled spectra with spectral variations of different bandwidths at 1.5, 3.0, and 5.0 c/o, respectively. Although the spectral shapes of these stimuli differed substantially (see Fig 2), the elevation response distributions were nevertheless quite similar. As the 1.5 c/o spectra contained considerably less spectral variation within and across neighbouring auditory filters than the other two stimulus sets, these stimuli might have been localized more accurately (i.e. more responses would be directed towards the veridical speaker location at zero degrees). However, we observed no systematic differences in the standard deviations of response elevation distributions for the three stimulus sets (see Table 1), indicating that for all three spectral variations, listeners made similarly large, and consistent mislocalizations away from the actual speaker position at [*α*, *ϵ*] = [0, 0].

### Effect of pre-knowledge?

Although we instructed our listeners to disregard the known veridical location of the speaker, and to respond to the perceived location, which could differ substantially, one may wonder whether this preknowledge may have somehow affected the localization responses nonetheless. In our experience, subjects are well able to accurately respond to perceived sound locations in the absence of any visual, acoustic, or verbal feedback (open-loop response paradigms). However, we cannot exclude the possibility of a cognitive influence in these spatial-illusion experiments, and there is a hint that this may indeed have played a small role in the responses of listener S2 (Fig 3B). For example, the implicit wish to avoid the (remembered) location at straight ahead could have induced a slight response bias away from that location. As a result, if the sound was perceived slightly below/above the horizon, responses could thus have overestimated the perceived location. Such a strategy would lead to a bimodal response distribtion, which would nearly exlcude the straight-ahead location. This was indeed observed in the responses of S2, but in none of the other listeners. However, if anything, such use of preknowledge would have had a detrimental influence on the spectral-shape reconstructions, as the straight-ahead location at zero elevation would become underrepresented. In Fig 11A this may indeed be apparent from the small gap around zero deg elevation in the reconstructed notch (the gray band running from 5–8 kHz), which was not observed in the reconstruction of the other listeners (see, e.g. the results of S4 in Fig 8A and 8B). This particular effect of preknowledge, however, resulted to be confined to locations close to straight ahead only, as the reconstructed spectral features for other elevation angles were not systematically affected, neither in listener S2, nor in S4. Note that the strength of the effect was reduced in our reconstructions, as we replaced every response by a Gaussian probability distribution around the true response (Methods). For that reason, the zero-degrees elevation angle contained some power in the reconstructions of listener S2, even though very few responses were directed to precisely that location.

That all listeners were unable to ignore the influence of the straight-ahead speaker location, however, is better explained by acoustic weighted averaging, than by a cognitive influence of pre-knowledge (the inability of ‘not responding to 0 at will’). Indeed, it was unavoidable that the speaker imprinted a strong HRTF from zero elevation on the sensory spectrum, together with the stimulus-induced random spectral features. Bremen et al. [17] have demonstrated that when two sound sources are presented synchronously in elevation, the localization response is unavoidedly a weighted average of the two perceived single-source elevations, in which the relative sound levels serve as weights. In the current experiments we cannot know a-priori how many potential elevation sources might have been (partly) present in the sensory spectrum, but we expect that a similar center-of-gravity weighting scheme would underlie the resulting perceived elevation. Such a weighting would be underlying cause of the reduced elevation span of the responses, as long as there is a strong influence of the zero-degree HRTF.

We assert that the high correlations between the first and second response to the randomly presented spectral shapes can only be explained by an acoustic processing mechanism. Whether or not subjects would be aware of the actual sound location, will not make a qualitative difference in their response behavior. At worst, if a listener would be totally unable to supress this preknowledge, she would only respond to the known straight-ahead location, thus rendering the reconstruction method used of this paper entirely useless. The finding that our reconstructions yielded a good resemblance with the measured DTFs for all subjects indicates that the potential influence of cognitive factors on these results was minor at best.

### Sound localization models

In localizing sounds in elevation, the auditory system has to deal with two unknown factors: **(i)** the contribution of the actual sound-source spectrum to the sensory spectrum, and **(ii)** the actual direction-dependent filter applied by the pinna. Middlebrooks [15] posed that in order to solve this ill-posed problem, the auditory system assumes that the source spectrum is broadband and flat, so that any spectral coloring can be assigned to the HRTF of the source direction. However, this would considerably restrict the type of sounds that can be localized accurately.

Zakarauskas and Cynader [18] therefore suggested a more relaxed assumption, in which either the source spectrum should be locally flat, or the slope of the spectrum should be locally constant, to ensure accurate localization, as the spectral changes imposed by the HRTF are rather steep. For the present experiments, this would imply that the stimuli with a bandwidth of 1.5 c/o should be localized more accurately (i.e., create more spatial illusions, and over a wider range) than the stimuli with a bandwidth of 5.0 c/o, as for the former stimuli the spectra contained markedly less variation (e.g., Fig 2). Contrary to this prediction, however, the standard deviations of the responses for the three stimulus sets were comparable (Fig 4).

To model sound elevation localization, Butler and colleagues introduced the concept of covert peak areas ([11], [12], [13], [14]): the area in space for which a narrow-band sound is amplified most. They posed that sounds are localized on the basis of the frequency segment with the maximum peak in the sensory spectrum (the covert peak). This suggests that only one peak per frequency segment is relevant for elevation localization. If this would be true, in our reconstructed spectra also only one peak should appear per frequency segment, as all other features would be cancelled out. Fig 6 shows that the reconstructed perceptual spectra show multiple peaks and notches for each frequency segment and for each elevation.

Hofman and Van Opstal [10] proposed a spectral cross-correlation model that makes no a-priori assumptions about the shape of the source spectrum. In their model, the sensory spectrum is compared to a library of neurally stored HRTFs for all directions. Two basic assumptions underly the model: the first is that HRTFs are unique, and do not resemble each other. This can be readily tested by correlating the HRTFs with each other. Such an analysis shows that HRTFs indeed contain unique information about the sound-source’s elevation (see e.g. [7], [10]). The second assumption is that (natural) free-field sound spectra do not correlate with any of the HRTFs in this stored representation. If true, it can be readily shown that the correlation between the sensory spectrum (the result of Eq 2) and the library of stored HRTFs will always peak at the veridical elevation angle of the sound source. The simplest version of the cross-correlation model would therefore be to find the peak in the cross-correlation, and to assign the perceived elevation to the location of that peak.

**Fig 12:**
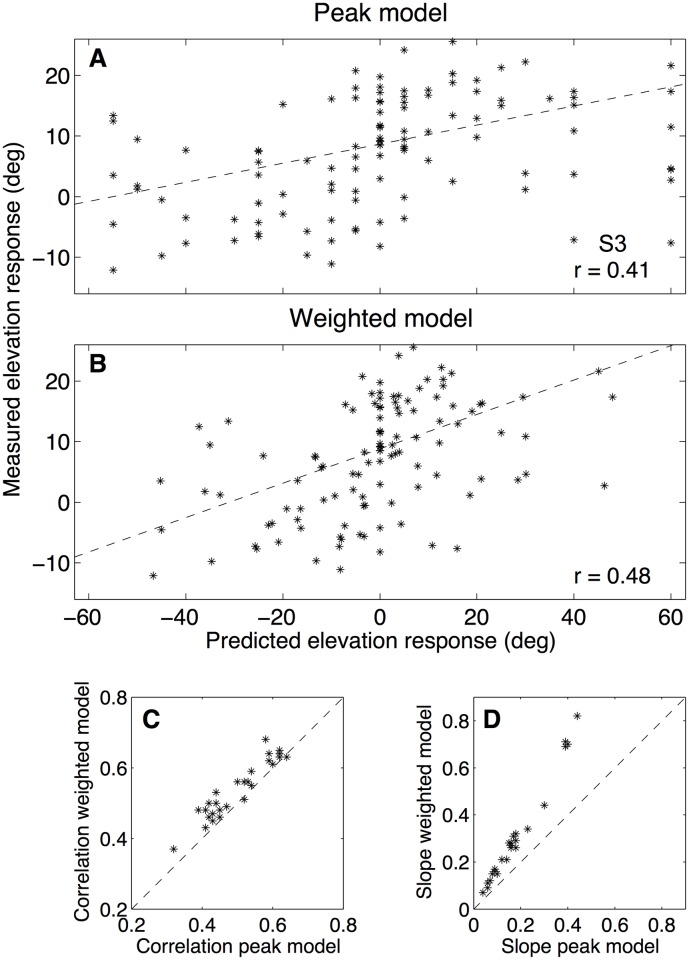
Improved model. **A:** Measured elevation responses vs. predicted elevation responses according to the maximum correlation model of Hofman and Van Opstal [10]). The correlation is indicated in the lower-right corner. Data for the 3.0 c/o stimuli of listener S3. **B:** Same data, predicted elevation responses according to the weighted correlation model (see text). **C:** Correlation values between measured and predicted elevation responses for all listeners and all stimulus sets. Values for the weighted correlation model are plotted against the values for the peak correlation model. Note that all data points are at or above the unity line. **D:** Same as in C but for the slope of the regression line through the measured vs. predicted elevation responses. Note that all data points are above the unity line.

To test this idea for the current experiments with random spectra, we calculated the spectral correlations between the sensory spectra of all stimuli (Eq 6) and all measured DTFs over a 4–14 kHz bandwidth for each of the listeners, and each of the three stimulus sets. In this frequency range one encounters the most prominent first-order direction-dependent peaks and notches of the DTFs for elevations within the frontal hemifield (the locations of second- and higher-order peaks and notches were typically more noisy, as they depend more on the exact location of the probe microphone within the ear canal). We then determined the elevation for which the correlation reached the maximum value, and compared this predicted elevation to the actual response of the listener. Note that this analysis does not account for the potential weighted averaging behavior of the auditory system in case the sensory spectrum contains multiple peaks (see also above; [17]). Fig 12A shows the measured response elevations vs. the predicted response elevations together with a regression line and the correlation for listener S3 for the 3.0 c/o stimuli. Although the slopes of the regression lines were generally low (mean value of 0.17 over all listeners and stimulus sets), correlations were all highly significant (*p* < 0.01), varying between 0.32 and 0.64 (mean: 0.50). Thus, the model does a reasonable job in explaining the variation of the observed behavior.

Nonetheless, the applied prediction is too simple to account for the observed responses, as it clearly falls short in explaining the observed resolution (i.e. the slope). There are at least two important points, not accounted for by this simple algorithm: first, rippled amplitude spectra are likely to yield several peaks in the DTF cross-correlations. In our experiments, the DTF corresponding to the straight-ahead speaker position had a large influence on the sensory spectrum, and therefore always induced a substantial peak in the cross-correlation. The presence of this peak could explain why the range of the listeners’ responses was compressed toward the straight-ahead direction, but none of the models predicts the amount by which such a compression should occur. The study of Bremen et al. [17] clearly indicated that the auditory system applies a spatial weighted average of the elevation angles, whenever the system is confronted with synchronous sound sources at different locations in the median plane. Presumably, the subject’s responses to the random spectral shapes in our experiments were determined by such an averaging mechanism too. Second, the simple correlation model works well if the stimulus does not correlate with the HRTFs *at all*, since in that case the peak in the cross-correlation will have a height close to one at the veridical elevation. However, the random spectral-shape stimuli applied in this study yielded relatively low correlations with the DTFs (values typically around 0.40, or less, not shown). The value of this correlation could be indicative to the auditory system for the reliability of that particular elevation angle, and may hence determine the willingness of the audiomotor system to move away from its default (prior) horizon (e.g. a preset bias, as seen in the median values in the response data of Table 1).

In a recent study we proposed that the value of this correlation, together with other sources of information, like pre-knowledge of the acoustic environment, might partially determine the gain of the elevation responses [27]. Interestingly, several studies have indicated that a systematic variation of acoustic parameters may lead to a systematic change of the slope of the stimulus-response relationship for elevation. For example, reducing the duration of a noise burst systematically reduces the elevation gain ([10] [27]). Changes of pinna shape through an experimental manipulation ([6] [7]), or through gradual pinna growth throughout life [8], induces shifts of the spectral cues to which the auditory system learns to adapt its stimulus-response relation. In addition, varying the stimulus level for short-duration stimuli leads to a variation of the slopes of the stimulus-response relation ([27], [28], [29]). Also the introduction of a noisy background, to manipulate the overall signal-to-noise ratio of the stimulus, systematically reduces the elevation response gain ([30], [31]). These acoustic manipulations have in common that they all affect the reliability of the spectral cues, albeit in different ways: background noise masks the spectral peaks and notches; loud, short-duration clicks may saturate the cochlear response, and hence abolish spectral detail, and soft brief noise bursts may deny the auditory system sufficient time to integrate acoustic input to extract adequate fine spectral detail.

Thus, a first extension of the original cross-correlation model [8] should incorporate a mechanism that also weighs the reliability of the spectral-shape estimate for a given elevation, for example, by calculating the center-of-gravity of the (positive) cross-correlation values of the sensory spectrum across multiple DTFs, rather than just selecting the site of maximum correlation:

$$\varepsilon_{perc}=\frac{\sum_{n}\;\rho_{n}\cdot \varepsilon_{n}}{\sum_{n}\;\rho_{n}}\;\;\text{for}\;\rho_{n}>\rho_{min}$$ (9)

where *ρ*_*n*_ (restricted to *ρ_min_* > 0.3) is the value of the spectral correlation between the sensory spectrum and the DTF for elevation angle *ε*_*n*_ (Eq 7).

The result of applying this analysis to the data of Fig 12A is illustrated in Fig 12B. Both the correlation between the predicted and measured elevation responses and the slope of the regression line increased; while the correlation improved significanty, the value for the slope almost doubled. This finding was representative for all listeners: on average the correlation improved from 0.50 to 0.54, whereas the mean slope increased from 0.17 to 0.30. In Fig 12C and 12D the correlation and slope of this weighted correlation model are plotted against the values for the peak-correlation model for all listeners and all stimulus sets. Note that virtually all data points lie above the unity line, indicating that this simple extension already improves the predictions of the simple cross-correlation model. Further support for the influence of multiple elevation peaks in the cross-correlation results followed from our experiment to move the speaker towards the zenith, which had a dramatic effect on the elevation response range of the listener (Fig 10).

## Conclusion

Taken together, we conjecture that the auditory system incorporates the possibility of multiple peaks in the cross-correlation functions, by weighting the different peaks (i.e. candidate elevation angles) by their reliability to calculate the perceived elevation. One possible weighting scheme could rely on taking the center of gravity of the potential elevations, in which the correlations between the stimulus spectrum and the DTFs act as weighting factors. Since in the present experiment the DTF for straight ahead would typically yield the highest correlation for most stimuli, a reduced elevation response range would result, just as observed in our subjects and [19].

Other, more elaborate models could be based on a true Bayesian mechanism in the auditory system, in which the weighting may also depend on a different prior distribution of spectral ripples than the straightforward uniform assumption in our current analyses (maximum likelihood estimation). This prior may be influenced by past experience, pre-knowledge of the speaker position, and/or by expectations on the distribution of spectral shapes. Further work will be needed to study these different possibilities.

## References

[1] Blauert J (1997) ‘Spatial Hearing. The Psychophysics of Human Sound Localization’, 2nd edition, Cambridge MA: MIT Press

[2] Middlebrooks JC and Green DM (1991) ‘Sound localization by human listeners.’ *Annu Rev Psychol* 42:135–159 10.1146/annurev.ps.42.020191.001031 2018391

[3] Takemoto H, Mokhtari P, Kato H, and Nishiura R (2012) ‘Mechanism for generating peaks and notches of head-related transfer functions in the median plane’, *J Acoust Soc Am* 132, 383–3841 10.1121/1.4765083 23231113

[4] Wightman FL and Kistler DJ (1989a) ‘Headphone simulation of free-field listening. I: Stimulus synthesis’, *J Acoust Soc Am* 85: 858–867 10.1121/1.397557 2926000

[5] Van Opstal AJ (2016) ‘The auditory system and human sound-localization behavior’, 1st Ed, Academic Press, Elsevier Publishers, Amsterdam, NL

[6] Hofman PM, Van Riswick JGA, and Van Opstal AJ (1998) ‘Relearning sound localization with new ears.’ *Nat Neurosci* 1: 417–421 10.1038/1633 10196533

[7] Van Wanrooij MM and Van Opstal AJ (2005) ‘Relearning Sound Localization with a New Ear.’ *J Neuroscience*, 25: 5413–5424 10.1523/JNEUROSCI.0850-05.2005 15930391 PMC6724994

[8] Otte RJ, Agterberg MJH, Van Wanrooij MM, Snik AFM and Van Opstal AJ (2013) ‘Age-related hearing loss and ear morphology affect vertical, but not horizontal, sound-localization performance.’ *JARO*, 14: 261–273 10.1007/s10162-012-0367-7 23319012 PMC3660912

[9] Zwiers MP, Van Opstal AJ and Paige GD (2003) ‘Plasticity in human sound localization induced by compressed spatial vision’, *Nat Neurosci* 6: 175–181 10.1038/nn999 12524547

[10] Hofman PM and Van Opstal JA (1998) ‘Spectro-temporal factors in two-dimensional human sound localization’, *J Acoust Soc Am* 103: 2634–2648 10.1121/1.422784 9604358

[11] Musicant AD and Butler RA (1984) ‘The psychophysical basis of monaural localization’, *Hear Res* 14: 185–190 10.1016/0378-5955(84)90017-0 6746432

[12] Butler RA (1987) ‘An analysis of the monaural displacement of sound in space’, *Percept Psychophys* 41: 1–7 10.3758/BF03208206 3822738

[13] Butler RA and Musicant AD (1993) ‘Binaural localization: influence of stimulus frequency and the linkage to covert peak areas’, *Hear Res* 67: 220–229 10.1016/0378-5955(93)90250-5 8340275

[14] Rogers ME and Butler RA (1992) ‘The linkage between stimulus frequency and covert peak areas as it relates to monaural localization’, *Percept Psychophys* 52: 536–546 10.3758/BF03206715 1437486

[15] Middlebrooks JC (1992) ‘Narrow-band sound localization related to external ear acoustics’, *J Acoust Soc Am* 92: 2607–2624 10.1121/1.404400 1479124

[16] Kulkarni A and Colburn HS (1998) ‘Role of spectral detail in sound-source localization’, *Nature* 396: 747–749 10.1038/25526 9874370

[17] Bremen P, Van Wanrooij MM, and Van Opstal AJ (2010) ‘Pinna cues determine orienting response modes to synchronous sounds in elevation’. *J Neurosci* 30: 194–204 10.1523/JNEUROSCI.2982-09.2010 20053901 PMC6632510

[18] Zakarauskas P and Cynader MS (1993) ‘A computational theory of spectral cue localization’, *J Acoust Soc Am* 94: 1323–1331 10.1121/1.408160

[19] Hofman PM and Van Opstal JA (2002) ‘Bayesion reconstruction of sound localization cues from responses to random spectra’, *Biol Cybern* 86: 305–316 10.1007/s00422-001-0294-x 11956811

[20] Collewijn H, Van Der Mark F, Jansen TC (1975) ‘Precise recording of human eye movements’, *Vision Res* 15: 447–450 10.1016/0042-6989(75)90098-X 1136166

[21] Knudsen EI and Konishi M (1979) ‘Mechanisms of sound localization in the barn owl (Tyto alba)’, *J Comp Physiol* 133: 13–21 10.1007/BF00663105

[22] Schroeder MR (1970) ‘Synthesis of low-peak factor signals and binary sequences with low autocorrelation’, *IEEE Trans Inf Theory* 16: 85–89 10.1109/TIT.1970.1054411

[23] Van Barneveld DCPBM, Van Grootel TJ, Alberts B, and Van Opstal AJ (2011) ‘The effect of head roll on perceived auditory zenith.’ *Exp Brain Res* 213: 235–243 10.1007/s00221-011-2741-9 21643715 PMC3155039

[24] Algazi VR, Avendano C, Duda RO (2001) ‘Elevation localization and head-related transfer function analysis at low frequencies’, *J Acoust Soc Am* 109: 1110–1122 10.1121/1.1349185 11303925

[25] Wightman FL and Kistler DJ (1989b) ‘Headphone simulation of free-field listening. II: Psychophysical validation’, *J Acoust Soc Am* 85: 868–878 10.1121/1.397558 2926001

[26] Makous JC and Middlebrooks JC (1990) ‘Two-dimensional sound localization by human listeners’, *J Acoust Soc Am* 87: 2188–2200 10.1121/1.399186 2348023

[27] Vliegen J, Van Opstal AJ (2004) The influence of duration and level on human sound localization’, *J Acoust Soc Am* 115: 1705–1713 10.1121/1.1687423 15101649

[28] Hartmann WM and Rakerd B (1993) ‘Auditory spectral discrimination and the localization of clicks in the saggital plane’, *J Acoust Soc Am* 94: 2083–2092 10.1121/1.407481 8227750

[29] MacPherson EA and Middlebrooks JC (2000) ‘Localization of brief sounds: Effects of level and background noise’, *J Acoust Soc Am* 108: 1834–1849 10.1121/1.1310196 11051510

[30] Good MD and Gilkey RH (1996) ‘Sound localization in noise: the effect of signal-to-noise ratio’, *J Acoust Soc Am* 99: 1108–1117 10.1121/1.415233 8609294

[31] Zwiers MP, Van Opstal AJ and Cruysberg JRM (2001) ‘A spatial hearing deficit in early-blind humans’, *J Neurosci* 21: RC142: 1–5 11312316 10.1523/JNEUROSCI.21-09-j0002.2001 PMC6762556

